# Clinical and Prognostic Characteristics of Recurrent Intracerebral Hemorrhage: A Contrast to First-Ever ICH

**DOI:** 10.3389/fnagi.2022.860571

**Published:** 2022-04-14

**Authors:** Yan Wan, Hongxiu Guo, Rentang Bi, Shaoli Chen, Jing Shen, Man Li, Yuanpeng Xia, Lei Zhang, Zhou Sun, Xiaolu Chen, Zhuoyuan Cai, Zhaowei Wang, Daokai Gong, Jingwen Xu, Dongya Zhu, Bo Hu, Quanwei He

**Affiliations:** ^1^Department of Neurology, Union Hospital, Tongji Medical College, Huazhong University of Science and Technology, Wuhan, China; ^2^Department of Neurology, Qianjiang Central Hospital, Qianjiang, China; ^3^Department of Neurology, Jingzhou Central Hospital, Jingzhou, China; ^4^Department of Neurology, Honghu People’s Hospital, Honghu, China; ^5^School of Pharmacy, Nanjing Medical University, Nanjing, China

**Keywords:** intracerebral hemorrhage, recurrence, clinical characteristics, prognosis, contrast

## Abstract

This study aimed to compare clinical and prognostic characteristics between recurrent and first-ever ICH. Four thousand twelve patients entered the study, and 64% of them were male. The median age is 62 years (interquartile range, 55–71). Among them, 3,750 (93.5%) patients had no experience of previous ICH, and 262 (6.5%) patients were considered as recurrent ICH. We compared demographic data, baseline clinical characteristics, imaging information, hematological parameters, and clinical outcomes between recurrent and first-ever ICH. We found that recurrent ICH was significantly associated with older age, more frequent history of ischemic heart disease, ischemic stroke, hypertension, and hyperlipidemia, while patients with recurrent ICH had previously received more antihypertensive therapy, and showed lower admission blood pressure (median, 160 vs. 167 mmHg) and higher baseline of National Institute of Health stroke scale (NIHSS) score (median, 10 vs. 9). We also demonstrated that recurrent ICH was an independent risk factor of 3-month function dependence after adjusting for many potentially competitive risk factors.

## Introduction

Intracerebral hemorrhage (ICH) is a more devastating disease than ischemic stroke, features a high mortality rate of 25–50% within a month (Lozano et al., 2012; Li et al., 2021), and the survivors remain at high risk for recurrence. Currently, ICH recurrence rates have been reported up to 2% in 1 year, and 9.6% in 5 years (Hill et al., 2000; Huhtakangas et al., 2013), and recurrent ICH seems to be more disabling or fatal than first-ever ICHs (Pennlert et al., 2014; Skajaa et al., 2021).

Despite various studies that have been conducted to search for candidate risk factors of ICH recurrence (Biffi et al., 2020, 2021; Miki et al., 2020; de Courson et al., 2021; Park et al., 2021a,b; Pinho et al., 2021), the clinical and prognostic characteristics of recurrent ICH have only received limited attention. Indeed, a reliable analysis of recurrent ICH is urgently required to guide the management strategies for secondary prevention and assess the cost-effectiveness of treatment, thereby improving the prognosis of patients. Importantly, controversy still surrounds whether recurrent ICH should be considered as an independent risk factor for adverse outcomes, because recurrent ICH may exhibit a distinguished baseline patient characteristics from first-ever ICH.

To this end, we conducted a population-based cohort study using a nationwide representative sample from the Chinese cerebral hemorrhage: mechanism and intervention (CHERRY) study, to compare clinical and prognostic characteristics between recurrent and first-ever ICH.

## Materials and Methods

### Study Population

We use a representative sample from the CHERRY study. This study included 4,012 patients in 31 medical institutions from December 2018 to March 2021. The ethics of the study is in line with the principles expressed in the Declaration of Helsinki. The local institutional review board approved all aspects of the study (ethical approval number: 2018-S485).

### Inclusion and Exclusion Criteria

Patients were included according to the following criteria: (1) clinically confirmed spontaneous ICH, which is defined as non-traumatic bleeding into the brain parenchyma confirmed by CT scan (de Oliveira Manoel et al., 2016); (2) 18 years or older; (3) the onset to admission time within 7 days. Exclusion criteria constituted: (1) traumatic ICH; (2) ICH colocalized with primary subdural/epidural/subarachnoid hemorrhage; (3) post-infarct hemorrhagic transformation; (4) hemorrhage after thrombolysis.

### Data Collection

The following data were collected: (1) demographic data, including age, gender, medical history, medication history (prior use of antithrombotic and antihypertensive agents); (2) admission data, including baseline blood pressure, the modified Rankin Scale (mRS), the National Institute of Health stroke scale (NIHSS), and the Glasgow Coma Score (GCS), imaging data, such as hematoma location, hematoma volume, and intraventricular hemorrhage (IVH); (3) hematological parameters; (4) the structural lesions, medication, amyloid angiopathy, systemic disease, hypertension, and undetermined (SMASH-U) etiology of ICH. Recurrent ICH was defined as any ICH ≥ 24 h after the first incident event (Coulland Rothwell, 2004). All recurrent ICHs were symptomatic with a newly occurred focal neurologic deficit or decreased level of consciousness, vomiting, headache, etc. The CT scan was performed to confirm the diagnosis of recurrent ICH. Medication history was defined as taking antithrombotic (antiplatelet or anticoagulation) or antihypertensive agents within 30 days before hospitalization for ICH. Alcohol drinking refers to patients who drink regularly, with more than one unit of alcohol (equals to 360 ml of beer, or 50 ml of white wine, or 120 ml of red wine) a week. Experienced neurologists performed the imaging analyses based on the initial CT scan, and hematoma volume was calculated using the ABC/2 formula.

### Clinical Outcomes

The primary outcome is death and functional dependence referred to the mRS score of 3–6 at 90 days. The second outcomes are 30-day functional dependence, and in-hospital, 30-day and 90-day death.

### Statistical Analysis

The continuous or discrete variables were presented as median with interquartile, and categorical variables were presented as percentages. Univariate analysis was analyzed using the χ2 test and Mann–Whitney *U* test for categorical variables and continuous variables, respectively. Non-normally distributed continuous variables were categorized based on clinical and statistical significance in the subgroup analysis. All variables of univariate analysis with a *P*-value < 0.1 were included in the multivariate regression model. All tests were two-tailed and a *P*-value < 0.05 was considered significant. Statistical analyses were performed using SPSS software (version 27.0) and R software (version 4.1.2).

## Results

### Baseline Characteristics of Participating Subjects

**FIGURE 1:**
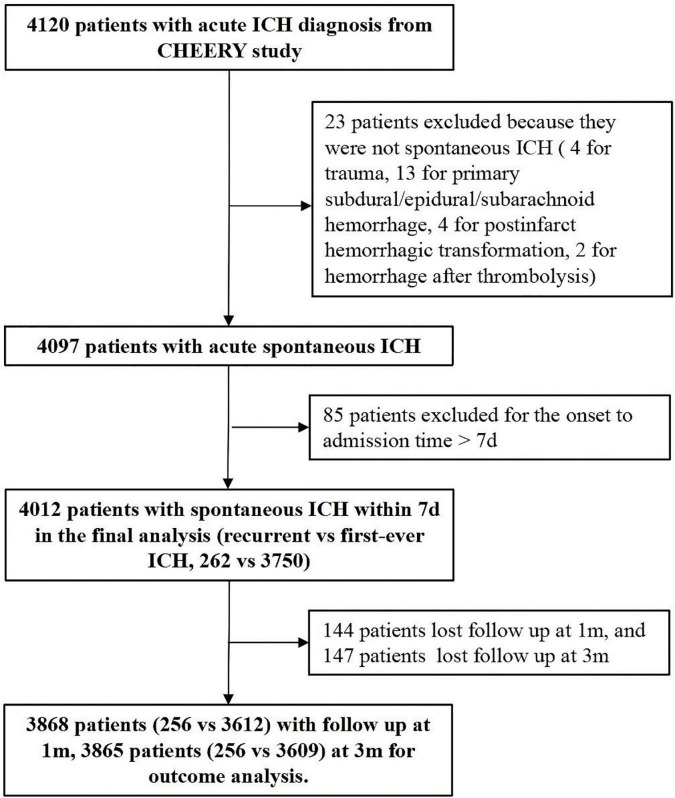
The patient enrollment flowchart.

After exclusion for ineligible patients, a total of 4,012 patients were entered into the study (see the patient enrollment flowchart in Figure 1). Among them, 64% of patients were male and the median age is 62 years (interquartile range, 55–71). Three thousand seven fifty (93.5%) patients had no experience of previous ICH, and 262 (6.5%) patients were considered as recurrent ICH. The baseline clinical characteristics of patients grouped by recurrent and first-ever ICH are summarized in Table 1.

**TABLE 1:** Comparisons of demographics and clinical characteristics between patients with first-ever ICH and recurrent ICH.

Characteristics	Recurrent ICH	First-ever ICH	*P*-value
Total No.	262	3750	
Age, years	63 (55–71)	62 (53–70)	0.023
Male	165 (63.0)	2543 (67.8)	0.106
**Medical history**			
Smoking	62 (23.7)	1183 (29.4)	**0.035**
Alcohol drinking	45 (17.2)	952 (23.7)	**0.010**
Ischemic heart disease	3 (1.1)	147 (3.9)	**0.022**
Ischemic stroke	38 (14.5)	346 (9.2)	**0.005**
Hypertension	212 (80.9)	2348 (62.6)	**<0.001**
Diabetes	18 (6.9)	337 (9.0)	0.244
Hyperlipidemia	13 (5.0)	91 (2.4)	**0.013**
**Medication history**			
Previous statin	5 (1.9)	51 (1.4)	0.465
Previous antithrombotic agent	12 (4.6)	166 (4.4)	0.907
Previous antihypertensive agent	58 (22.1)	582 (15.5)	**0.005**
**Admission vitals**			
Baseline mRS	4 (2–5)	4 (2–5)	0.081
Baseline NIHSS	10 (4–20)	9 (3–15)	**0.011**
Baseline GCS	14 (9–15)	14 (10–15)	0.883
Baseline SBP, mm Hg	160 (145–177)	166 (148–186)	**0.002**
**Admission laboratory values**			
Leukocyte, ×10^9^/L	8.6 (6.5–10.8)	8.5 (6.5–11.3)	0.487
Hemoglobin, g/L	136 (123–146)	135 (123–147)	0.714
Platelet, ×10^9^/L	195 (155–239)	193 (153–237)	0.618
International normalized ratio	1.0 (1.0–1.1)	1.0 (0.9–1.1)	**0.001**
Prothrombin time, s	12.2 (11.1–13.1)	12.0 (11.0–13.1)	0.289
Activated partial thromboplastin time, s	28.8 (25.5–33.1)	28.6 (24.6–32.8)	0.245
Alanine transaminase, U/L	17.0 (12.2–24.0)	18.0 (13.0–26.0)	**0.04**
Aspertate aminotransferase, U/L	22.0 (17.0–29.0)	23.0 (18.0–30.0)	0.147
Creatinine, μmol/L	68.4 (56.1–87.5)	68.6 (56.4–84.0)	0.473
Total cholesterol, mmol/Lb	4.4 (3.7–5.1)	4.5 (3.9–5.2)	**0.03**
Total triglycerides, mmol/L	1.1 (0.8–1.5)	1.2 (0.9–1.8)	**0.005**
High density lipoprotein cholesterin, mmol/L	1.2 (1.0–1.4)	1.3 (1.0–1.6)	**0.001**
Low density lipoprotein cholesterin, mmol/L	2.5 (2.1–3.2)	2.6 (2.1–3.2)	0.325
Fasting blood-glucose, mmol/L	6.2 (5.4–7.7)	6.3 (5.3–7.8)	0.567
**Admission imaging data**			
Location of hematoma			
Lobar	46/249 (18.5)	631/3606 (17.5)	0.922
Non-lobar	191/249 (76.7)	2804/3606 (77.8)	
Mixed	12/249 (4.8)	171/3606 (4.7)	
Supratentorial	208/249 (83.5)	3103/3606 (86.1)	0.285
Infratentorial	36/249 (14.5)	465/3606 (12.9)	
Mixed	5/249 (2.0)	38/3606 (1.1)	
Hematoma volume, mL	10.0 (4.8–25.0)	12.0 (5.1–27.0)	0.270
Intraventricular hemorrhage	50 (19.1)	688 (18.3)	0.766
**SMASH-U etiology**			
Structural lesion	12 (4.6)	205 (5.5)	**<0.001**
Systemic disease	10 (3.8)	188 (5.0)	
Medication	2 (0.8)	19 (0.5)	
Amyloid angiopathy	37 (14.1)	511 (13.6)	
Hypertensive angiopathy	154 (58.8)	1707 (45.5)	
Undetermined	47 (17.9)	1120 (29.9)	

*Bold values represent P < 0.05.*

In contrast to the first-ever ICH group, recurrent ICH patients were in older median age, having a more frequent history of smoking, alcohol drinking, ischemic stroke, ischemic heart disease, hypertension, and hyperlipidemia, accompanied with higher baseline NIHSS score. Meanwhile, recurrent ICH patients had previously received more antihypertensive therapy and featured lower admission blood pressure (median, 160 vs. 166 mmHg). Among admission laboratory tests, recurrent ICH was found related to lower alanine transaminase (ALT), total cholesterol (TC), total triglycerides (TG), high-density lipoprotein cholesterin (HDL-C), and international normalized ratio (INR). Moreover, ICH etiologies of amyloid angiopathy and hypertension were obsevered more frequently in recurrent ICH than first-ever ICH. However, there was no significant difference in the volume and location of a hematoma between the two groups.

### Recurrent Intracerebral Hemorrhage and Clinical Outcomes in Contrast to First-Ever Intracerebral Hemorrhage

**FIGURE 2:**
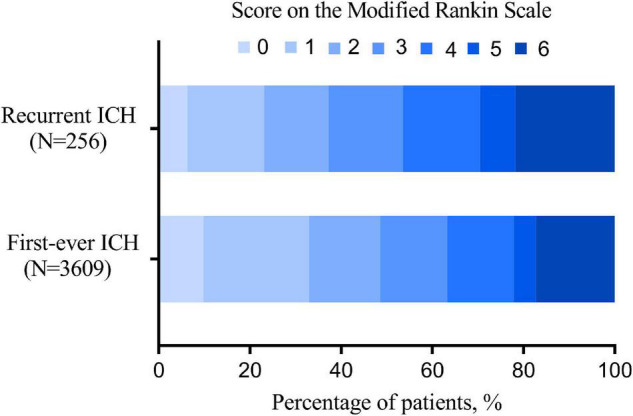
The distribution of 3-month modified Rankin Scale (mRS) of recurrent and first-ever intracerebral hemorrhage (ICH).

When clinical outcomes are compared between recurrent and first-ever ICH, recurrent ICH was associated with more 3-month functional dependence (Table 2), with the distribution of 3-month mRS, as provided in Figure 2. After adjusting for these competitive risk factors, including age, smoking, alcohol drinking, history of ischemic heart disease, ischemic stroke, hypertension, and hyperlipidemia, the previous antihypertensive agent, baseline mRS, and NIHSS, SMASH-U etiology, admission systolic blood pressure (SBP), ALT, TC, TG, HDL-C, and INR, the odds ratio (OR) of recurrent ICH was 1.545 (95% CI, 1.029-2.319) for functional dependence at 3 months.

**TABLE 2:** Odds ratio (OR) and 95% CI of Clinical Outcomes.

Clinical outcomes	First-ever ICH	Recurrent ICH	Unadjusting *P*-value	Adjusting *P*-value	Adjusting OR (95% CI)
**Primary outcome**					
Functional dependence at 3 m	1858/3609 (51.5)	161/256 (62.9)	<0.001	0.036	1.545 (1.029–2.319)
**Secondary outcomes**					
In-hospital death	152/3750 (4.1)	18/262 (6.9)	0.029	0.873	1.069 (0.471–2.427)
Functional dependence at 1 m	2167/3612 (60.0)	180/256 (70.0)	0.222	0.108	1.417 (0.927–2.168)
Death at 1 m	494/3612 (13.7)	42/256 (16.4)	0.001	0.591	1.152 (0.688–1.930)
Death at 3 m	617/3609 (17.1)	56/256 (21.9)	0.051	0.423	1.210 (0.758–1.932)

*Adjusting for these competitive risk factors, including age, smoking, alcohol drinking, history of ischemic heart disease, ischemic stroke, hypertension, and hyperlipidemia, previous antihypertensive agent, baseline mRS and NIHSS, SMASH-U etiology, admission SBP, ALT, INR, TC, TG, and HDL-C. OR, odds ratio.*

### Subgroup Analysis of the Association Between Recurrent Intracerebral Hemorrhage and Primary Outcome

We further conducted a subgroup analysis on the above logistic regression results. No statistically significant interaction between recurrent ICH and these interesting factors was observed (all *P*-values of interaction are greater than 0.05; Table 3).

**TABLE 3:** Subgroup analysis of the association between recurrent ICH and 3-month functional dependence.

Subgroup	3-month functional dependence		
	OR (95% CI)	*P*-value	*P* of interaction
**Age, years**			
<60	1.417 (0.858, 2.339)	0.173	0.353
≥60	1.975 (1.298, 3.006)	0.001	
**Sex**			
Male	1.953 (1.295, 2.943)	0.001	0.430
Female	1.449 (0.868, 2.419)	0.156	
**SBP, mmHg**			
<160	1.503 (0.955, 2.365)	0.078	0.456
≥160	1.937 (1.230, 3.052)	0.004	
**Admission NIHSS score**			
<10	1.766 (1.194, 2.612)	0.004	0.803
≥10	1.672 (0.966, 2.896)	0.066	
**Previous antihypertensive agent**			
No	1.793 (1.252, 2.569)	0.001	0.507
Yes	1.489 (0.716, 3.097)	0.287	
**Hypertension**			
No	1.341 (0.661, 2.720)	0.416	0.316
Yes	1.908 (1.328, 2.742)	<0.001	
**Dyslipidemia**			
No	1.724 (1.241, 2.394)	0.001	0.990
Yes	0.867 (0.130, 5.778)	0.867	
**Previous ischemic stroke**			
No	1.536 (1.089, 2.166)	0.014	0.087
Yes	4.079 (1.590, 10.456)	0.003	
**Previous ischemic heart disease**			
No	1.677 (1.214, 2.316)	0.002	0.405
Yes	16.994 (0.907, 318.586)	0.058	
**Current cigarette smoking**			
No	1.591 (1.097, 2.307)	0.014	0.244
Yes	2.308 (1.210, 4.403)	0.011	
**Current alcohol drinking**			
No	1.668 (1.169, 2.379)	0.005	0.627
Yes	1.998 (0.993, 4.278)	0.075	
**SMASH-U etiology**			
Structural lesion	2.447 (0.528, 11.341)	0.253	0.389
Systemic disease	9.617 (0.747, 123.881)	0.083	
Medication#	-	-	
Amyloid angiopathy	2.440 (1.008, 5.907)	0.048	
Hypertensive angiopathy	1.491 (0.979, 2.270)	0.063	
Undetermined	1.739 (0.840, 3.603)	0.136	

*Interactions between recurrent ICH and interesting factors on the primary outcome were tested by the likelihood ratio test with adjustment for variables in Table 1 unless the variable was used as a subgroup variable. NIHSS, National Institutes of Health Stroke Scale; OR, odds ratio.*

*#Subgroup analysis cannot be performed because the sample size is too small in the medication group.*

## Discussion

Our study showed that: (1) recurrent ICH was significantly associated with older age, more frequent history of ischemic heart disease, ischemic stroke, hypertension, hyperlipidemia, and higher baseline NIHSS score; (2) patients with recurrent ICH had previously received more antihypertensive therapy and showed lower admission blood pressure; (3) recurrent ICH was independently associated with poor 3-month functional outcomes.

Our analysis revealed that age, previous ischemic stroke, hypertension, and hyperlipidemia show significant group differences between recurrent and first-ever ICH patients. These are all the examined risk factors of ICH recurrence (Raffeld et al., 2015; Biffi et al., 2021; Pinho et al., 2021). Although age and medical history cannot be reversed, the risk of ICH recurrence may be reduced by preferable management strategies.

Intriguingly, patients with recurrent ICH exhibited even lower admission blood pressure, which may be attributable to the previous applications of the antihypertensive agents. Also, it is noteworthy that the admission SBP of patients with recurrent ICH was still as high as 160 (quartile, 145–177) mmHg, although it may be affected by an acute hypertensive response (Hawkesand Rabinstein, 2021). It is unclear whether this difference in blood pressure affects prognosis, as the recent, large, randomized, and controlled trials have not shown any benefit of intensive hypotension (≤160 mmHg) after ICH (Anderson and Qureshi, 2015; Qureshi et al., 2020). It may be speculated that blood pressure in patients, after the first ICH, has still not received enough attention. Effective management of hypertension should be pursued for the secondary prevention of ICH, especially in the Chinese population, which is characterized by suboptimal blood pressure control.

Moreover, our results revealed that patients with recurrent ICH showed lower total serum cholesterol, which has been considered inversely associated with ICH recurrence in previous studies (Koch, 2011; Baang and Sheth, 2021), although lipid control is often necessary for patients with ICH, concerns should remain whether intensive lipid-lowering is necessary for patients with recurrent ICH (Gurevitz et al., 2022). Meanwhile, the patients with recurrent ICH showed statistically lower ALT (17 vs. 18 U/L, *P* = 0.04), but ALT was almost within the normal range in both groups. This may be due to the improvement in liver function caused by the patient’s lifestyle optimizations after the cerebral hemorrhage, such as avoiding alcohol and high-fat diets.

Most importantly, the patients with recurrent ICH showed a higher risk of functional disability compared with first-ever ICH (62.9% vs. 51.5%, *P* < 0.001). Most of the previous articles only studied the recurrence of ICH as one of the clinical outcomes, while few studies specifically investigated the prognosis of recurrent ICH (Miki et al., 2020; Park et al., 2021a,b; Pinho et al., 2021; Skajaa et al., 2021), and the proportion of patients with ICH with 90-day adverse outcomes that they reported was 40–56% (Anderson et al., 2013; An et al., 2017; Fukuda-Doi et al., 2021). We firstly demonstrated that recurrent ICH is an independent risk factor for 3-month poor outcome after adjusting for potentially competitive risk factors, and this result showed consistency among SMASH-U etiological subtypes. This means that even if the risk factors, such as blood pressure and blood lipid, are perfectly controlled after the first ICH, the risk of ICH recurrence is still higher than that of the healthy population. However, the specific mechanism remains to be further explored.

Our study is original and has several strengths: (1) we conducted a national multi-center and large-sample study; (2) we strictly reviewed previous disease history for decades; (3) numerous confounders were adjusted to determine the association between ICH recurrence and clinical outcomes. After all, the limitations of our study lie in that: (1) lack of continuous monitoring to clinical characteristics, such as blood pressure and plasma glucose; (2) The enrolled patients were all Chinese.

## Data Availability Statement

The raw data supporting the conclusions of this article will be made available by the corresponding authors, without undue reservation.

## Ethics Statement

The studies involving human participants were reviewed and approved by Ethics Committee of Huazhong University of Science and Technology. The patients/participants provided their written informed consent to participate in this study. Written informed consent was obtained from the individual(s) for the publication of any potentially identifiable images or data included in this article.

## Author Contributions

YW, HG, and RB conducted the data analysis and wrote the manuscript. BH together with QH designed this study and directed the writing of the manuscript. SC, JS, ML, YX, LZ, ZS, XC, ZC, ZW, DG, JX, and DZ helped with the data collection and literature search. All authors contributed to the article and approved the submitted version.

## Conflict of Interest

The authors declare that the research was conducted in the absence of any commercial or financial relationships that could be construed as a potential conflict of interest.

## Publisher’s Note

All claims expressed in this article are solely those of the authors and do not necessarily represent those of their affiliated organizations, or those of the publisher, the editors and the reviewers. Any product that may be evaluated in this article, or claim that may be made by its manufacturer, is not guaranteed or endorsed by the publisher.
